# Chronic toxicity of shrimp feed added with silver nanoparticles (Argovit-4®) in *Litopenaeus vannamei* and immune response to white spot syndrome virus infection

**DOI:** 10.7717/peerj.14231

**Published:** 2022-11-22

**Authors:** Carlos R. Romo Quiñonez, Píndaro Alvarez-Ruiz, Claudio H. Mejía-Ruiz, Nina Bogdanchikova, Alexey Pestryakov, Carina Gamez-Jimenez, Wenceslao Valenzuela-Quiñonez, Magnolia Montoya-Mejía, Eusebio Nava Pérez

**Affiliations:** 1 Laboratorio de Biotecnología de Organismos Marinos, Centro de investigaciones Biológicas del Noroeste, La Paz, Baja California Sur, México; 2 Departamento de Acuacultura, Instituto Politécnico Nacional CIIDIR-Sinaloa, Guasave, Sinaloa, México; 3 Fisicoquímica de nanomateriales, Centro de Nanociencias y Nanotecnología, Universidad Nacional Autónoma de México, Ensenada, Baja California, México; 4 Research School of Chemistry & Applied Biomedical Sciences, Tomsk Polytechnic University, Tomsk, Russia

**Keywords:** Silver nanoparticles, Shrimp, Aquaculture, Chronic toxicity, WSSV, AgNP, Argovit, Litopenaeus vannamei, Silver fed, White spot syndrome virus

## Abstract

In recent years, the application of silver nanoparticles (AgNPs) as antibacterial compounds has been widely used in human and veterinary medicine. In this work, we investigated the effects of AgNPs (Argovit-4®) as feed additives (feed-AgNPs) on shrimp (*Litopenaeus vannamei*) using three different methods: 1) chronic toxicity after 28 days of feeding, 2) Effects against white spot syndrome virus (WSSV) challenged by oral route, and 3) transcriptional responses of immune-related genes (PAP, ProPO, CTL-3, Crustin, PEN3, and PEN4) following WSSV infection. The results showed that the feed-AgNPs did not interfere with the growth and survival of shrimp. Also, mild lesions in the hepatopancreas were recorded, proportional to the frequency of the feed-AgNP supply. Challenge test versus WSSV showed that feeding every 7 days with feed-AgNPs reduced mortality, reaching a survival rate of 53%, compared to the survival rates observed in groups fed every 4 days, daily and control groups of feed-AgNPs for the 30%, 10%, and 7% groups, respectively. Feed-AgNPs negatively regulated the expression of PAP, ProPO, and Crustin genes after 28 days of treatment and altered the transcriptional responses of PAP, ProPO, CTL-3, and Crustin after WSSV exposure. The results showed that weekly feeding-AgNPs could partially prevent WSSV infection in shrimp culture. However, whether or not transcriptional responses against pathogens are advantageous remains to be elucidated.

## Introduction

Worldwide shrimp farming (*Litopenaeus vannamei*) has grown from 2.6 million tonnes in 2010 to 4.9 million tonnes in 2018 (FAO, 2020). However, this growth has contributed to the degradation of farm ecosystems and the occurrence of diseases that affect production. In this sense, white spot disease (WSD), caused by the white spot syndrome virus (WSSV), is the most devastating disease worldwide, with a mortality rate of up to 100% in culture (Feng et al., 2017). Repeated pathogens outbreaks in shrimp farms have prompted research into new technologies to produce effective antimicrobial agents. Recently, silver nanoparticles (AgNPs) showed antiviral ability against WSSV in shrimp (Juárez-Moreno et al., 2017; Ochoa-Meza et al., 2019). The size and shape of AgNPs are associated with antimicrobial properties (Vaseeharan, Ramasamy & Chen, 2010; Morales-Covarrubias et al., 2016). Some formulations of AgNPs have been demonstrated in the clinical and veterinary fields (Dakal et al., 2016). AgNPs tend to self-aggregate, but dispersion is necessary to maintain antimicrobial effects; therefore, they must be surrounded by ligands that stabilize them (Ajitha et al., 2016). AgNPs showed antiviral activity against pathogenic viruses such as HIV-1 (Elechiguerra et al., 2005; Lara et al., 2010), hepatitis B virus (Lu et al., 2008), monkeypox virus (Rogers et al., 2008), herpes simplex virus type I (Baram-Pinto et al., 2009), tacaribe virus (Speshock et al., 2010), H1N1 influenza A virus (Mori et al., 2013), canine distemper virus (Bogdanchikova et al., 2016) and Rift Valley fever virus (Borrego et al., 2016). Recently, a single dose of AgNPs (called Argovit-4®) included in shrimp feed showed antiviral effects against WSSV (Romo-Quiñonez et al., 2020). However, there is no information on the effects of a regular supply of AgNPs in the feed.

The toxicity of AgNPs is related to shape, surface charge, size, dose delivered, and nanoparticle agglomeration state (Coutiño, Ávila Lagunes & Arroyo-Helguera, 2017). Acute toxicity is the effect of a single exposure to the agent. Instead, chronic toxicity depends on the agent’s persistence and the cells’ ability to remove it. Therefore, despite the antiviral effects of AgNPs, adverse effects in shrimp after treatment must be determined. The accumulation of metallic silver in marine invertebrates occurs mainly in the gills and hemolymph, and elimination is associated with the hepatopancreas (Bianchini et al., 2007). Intramuscular injection of 2 µg AgNP in *L. vannamei* caused no apparent damage to the shrimp (Juárez-Moreno et al., 2017). Romo-Quiñonez et al. (2020) added AgNPs (1000 µg AgNPs/g feed) to the feed for 8 days to feed shrimp; no adverse effects were found in the experimental shrimp.

Oxidative stress caused by accumulated reactive oxygen species (ROS) is the primary mechanism of AgNPs toxicity (Hsin et al., 2008). The antioxidant activity of shrimp is modulated by enzymes such as glutathione peroxidase (GPx), catalase (CAT), and superoxide dismutase (SOD). Those enzymes neutralize ROS-related oxidative stress (Liu, Tseng & Cheng, 2007). A balance must be maintained between organism’s reactive oxygen species and antioxidant activity. When increased ROS upsets this balance, oxidative stress causes cellular damage (Kohen & Nyska, 2002). AgNPs interfere with GPx and SOD enzymes, reducing their activity and promoting lipid peroxidation (Carlson et al., 2008). Therefore, oxidative stress caused by ROS accumulation can lead to many physiological and cellular imbalances, including mitochondrial destruction, apoptosis, inflammation, and DNA damage (Ahamed et al., 2010).

The shrimp’s hepatopancreas (HP) is the principal organ that carries out the digestive process. It is the first organ to receive the nutrients and components of all the substances it ingests, including contaminating elements such as heavy metals that can cause cellular damage (Wu & Yang, 2011). Therefore, histological analysis was performed in this study to determine cellular damage in shrimp HP cells after 28 days of AgNPs exposure.

The crustacean immune system is based on innate immunity, mediated by hemocytes, through cellular processes such as phagocytosis, encapsulation, and nodule formation (Söderhäll & Cerenius, 1992), synthesis of antimicrobial peptides (such as C-type lectins) or by prophenoloxidase mechanism (Tassanakajon et al., 2013). So far, it is unclear whether AgNPs affect shrimp’s immune defense against pathogens. Therefore, in this study, we assessed the transcriptional responses of genes related to cellular defense (phagocytosis-activating protein (PAP)) (Soto-Alcalá et al., 2019) and humoral defense (prophenoloxidase (ProPO), lectin 3 type C (CTL3), penaeidin 3 (Pen3), and crustin (Crustin)) (Qin et al., 2018; Soto-Alcalá et al., 2019; Soto-Alcalá et al., 2020), in experimental shrimp treated with feed-AgNPs in feed, and challenged against WSSV.

We found that feeding shrimp once a week with AgNPs (Argovit-4®) in the feed did not affect shrimp growth and helped counteract the effects of WSSV infection.

## Material and Methods

### Experimental organisms

*L. vannamei* shrimp were donated by Inmobiliaria Osiba, SA de CV, Sinaloa, Mexico. Shrimp were selected and transported to Interdisciplinary Research Center for Integrated Regional Development facilities in Sinaloa, Mexico. Shrimp were acclimated for a week in 1,000 L tanks with filtered seawater at 30 practical salinity units (PSU), 25 ± 2 °C and constant aeration.

Ten shrimp were analyzed by PCR with specific primers to verify that they were free of white spot syndrome virus (WSSV), infectious hypodermal and hematopoietic necrosis virus (IHHNV) or *Vibrio parahaemolyticus*, which is the causal agent of acute hepatopancreatic necrosis disease (VpAHPND), following the protocols described by Durand & Lightner (2002), Tang & Lightner (2001) and Han et al. (2015), respectively.

### Preparation of the experimental diet with AgNPs

According to a previous study (Romo-Quiñonez et al., 2020), Argovit-4® AgNPs were selected to prepare diets at a concentration of 1,000 µg AgNPs/g feed. 2 kg of commercial feed Camaronina® (Purina®, 35% protein) was pulverized to obtain flour. As an extruding agent, sodium alginate (2% w/w) (9005-38-3 Sigma-Aldrich®, St. Louis, MO, USA) was added. Subsequently, 166 ml of AgNPs (12 µg/mL) were diluted in 500 ml of distilled water and mixed with the flour (2 kg); after that, water was added until a homogeneous paste was obtained. Pellets were formed using a 50-ml syringe (no needle), the feed was dried in a refrigerator at 4 °C, and a control feed (without AgNP) was prepared in the same manner.

### WSSV-infected shrimp muscle for the challenge

Thirty shrimp (15 ± 2 g each) were injected intramuscularly with 100 µl of the WSSV inoculum each, between the 3rd and 4th abdominal segments. The inoculum was prepared from experimentally infected shrimp as previously described (Alvarez-Ruiz et al., 2013). After injection, shrimp were placed in aquariums with seawater at 30 PSU, 27 ± 1.0 °C, with constant aeration and a mechanical filter. Dying shrimp were collected 24 to 48 h post-infection (hpi); the head and exoskeleton was removed, and muscle tissue was stored at −70 °C.

Before the challenge test, the muscle tissue was thawed, weighed, and liquefied with seawater in a food processor (Nutribullet-600), filter through a 1.35 mm plastic mesh and add it immediately to the infection tank.

### Bioassay 1: chronic toxicity test

AgNPs included in the feed (Feed-AgNPs) were supplied in three different cycles for 28 days to assess the chronic toxicity of shrimp (4 ± 0.5 g). The experimental system comprised 12 tanks (150 L each) in a recirculating aquaculture system (RAS) filled with seawater at 30 PSU, filtered and chlorinated. Twenty shrimps were placed in each tank (three tanks per treatment), 5% of the body weight was fed daily, and the diets were divided into two rations (9:00 and 16:00 h) for 28 days. Feed-AgNPs were supplied once in the morning (2.5% of the body weight) in three different cycles: shrimp fed feed-AgNPs every day (D1); shrimp fed feed-AgNPs every four days (D4); shrimp fed feed-AgNPs every 7 days (D7), and the control group was fed without AgNPs (Ctrol). Temperature, dissolved oxygen (DO), and mortality were recorded daily. Growth, percent weight gain (WG), specific growth rate (SGR), feed conversion factor (FCR), and survival were recorded weekly. Diet performance was assessed by calculating percentage of body weight gain WG = [(final body weight − initial body weight)/initial body weight] × 100; specific growth rate SGR = 100 (ln average final weight × ln average initial weight)/days in culture; feed conversion ratio FCR = total dry feed intake (g)/wet weight gain (g); percentage of survival = (final number of shrimp/initial number of shrimp) × 100.

### Bioassay 2: challenge against WSSV

At the end of bioassay 1, shrimp were orally challenged with WSSV by providing infected shrimp muscle. A total of 40 shrimp from each treatment were placed in cages (5 mm plastic mesh) into a 100 L tank containing 40 L of seawater (30 PSU) at 27 ± 1 °C, and constant aeration. Just before the challenge, 40 g of infective shrimp muscle was thawed and processed as described above. After that, tissue was added to the infection tank, and after 12 h, shrimp were transferred to 40 L aquariums containing 30 L of seawater (10 shrimp per aquarium = four aquariums per treatment). Shrimp were fed *ad libitum*, organic waste was removed by siphoning daily, 50% water exchange was performed every two days, and mortality was recorded twice a day for 10 days. Dead or dying shrimp were removed from the aquarium and stored at −20 °C for later analysis. At the end of the experiment, three dead and three surviving shrimp from each treatment were analyzed by PCR to confirm WSSV status.

The fourth replicate of each treatment was used to assess transcriptional responses of immune-related genes.

### WSSV Detection

WSSV in shrimp was identified according to the method of Durand & Lightner (2002). DNA extraction from gill tissue was performed using DNAzol (MRC®, Cincinnati, OH, USA) following the manufacturer’s instructions. DNA was quantified in a NanoDrop 2000 (ThermoFisher Scientific, Waltham, MA, USA). A 260/280 nm absorbance ratio between 1.8 and 2.0 was considered adequate for PCR.

Each PCR reaction contains 1.5 µL 10X reaction buffer, 0.75 µL 50 mM MgCl_2_, 0.3 µL 10 mM dNTPs, 0.5 µL 10 µM each primer (forward/reverse), 1.15 µL 2.0 µM TaqMan Probe, 0.1 µL Recombinant Invitrogen® DNA polymerase (Life Technologies, Carlsbad, CA, USA), 100 ng DNA (sample), and ultrapure water to a final volume of 15 µL.

Amplification conditions were as follows: 95 °C for 3 min, followed by 40 cycles of 95 °C for 15 s, 60 °C for 30 s, and 72 °C for 20 s. Samples amplified after cycle 38 were considered negative.

### Bioassay 3

#### Transcriptional response of immune-related genes to WSSV infection

The expression of five genes encoding shrimp immune-related proteins was assessed: phagocytosis-activating protein (PAP) [cellular defense]; and prophenoloxidase (ProPO), C-type lectin 3 (CTL3), Penaeidin 3 (PEN3), Penaeidin 4 (PEN4), and crustin (Crustin (humoral system)). Transcriptional responses were assessed in shrimp hemocytes (*n* = 4) at different times: pre-infection (0 hpi) and 6 and 12 h post-infection (hpi).

### Hemolymph extraction

The hemolymph of individual shrimp (100–200 µl) was withdrawn from the ventral sinus of the first abdominal segment with a 1-mL syringe preloaded with 50 µl of anticoagulant (1X PBS (137 mM NaCl, 2.7 mM KCl, 10 mM Na_2_HPO_4_, 1.8 mM KH2PO_4_, pH 7.4) with 5% potassium oxalate w/v). Hemolymph was centrifuged at 800 × g for 10 min at 4 °C. The plasma was decanted, and the remaining plasma was removed with a micropipette. Finally, 200 µL of Trizol Reagent® was added to each sample and stored at −70 °C until RNA extraction.

### RNA extraction and cDNA synthesis

With some modifications, total RNA extraction was performed using Trizol reagent according to the manufacturer’s protocol. Hemocytes stored at −70 °C were homogenized with a pestle, and an additional 300 µl Trizol was added. After that, the manufacturer’s instructions were followed. In the end, RNA was diluted in 25 µl ultrapure water. Total RNA concentration and purity were measured in a NanoDrop 2000 (ThermoFisher scientific, Waltham, MA, USA). The RNA was treated with one unit of DNase I (1 U/µL; Sigma-Aldrich, St. Louis, MO, USA). cDNA synthesis was performed from 500 ng of total RNA using Improm II Reverse Transcriptase (Promega, Madison, WI, USA) and oligo dT20. The obtained cDNA was diluted with 80 µL of ultrapure water and stored at −70 °C until analysis. 5 µL of cDNA dilution was used as a template for qRT-PCR reactions.

### Quantitative RT-PCR analysis

**Figure 1:**
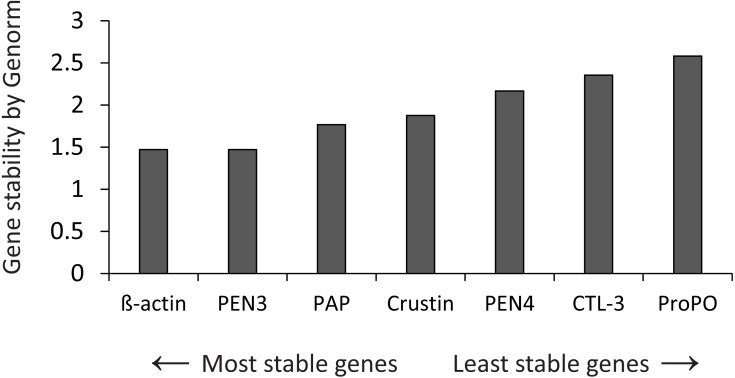
Gene stability by Genorm. Genorm analyzed the expression stability of all genes.

The stability of expression of the candidate housekeeping genes (Table 1) was analyzed by Genorm (Vandesompele et al., 2001), using the RefFinder web application (http://www.ciidirsinaloa.com.mx/RefFinder-master/?type=reference) (Fig. 1). The expression of target genes was normalized to the most stable gene (*β*-actin). Reactions were performed in a CFX96 real-time PCR thermal cycler (Bio-Rad Laboratories, Hercules, CA, USA) using 96-well plates.

**Table 1:** Primers used for qRT-PCR in this study.

**Primer**	**Sequence (5′** - **3′)**	**Product size (bp)**	**Reference**
PAP-F	CGAAGTTCAGGTTGTGCGTG	126	Soto-Alcalá et al. (2019)
PAP-R	ACTGATGCACCATTGGCCTT		
Crustin-F	GAGGGTCAAGCCTACTGCTG	157	Wang et al. (2010)
Crustin-R	ACTTATCGAGGCCAGCACAC		
proPO-F	CTGGGCCCGGGAACTCAAG	125	Soto-Alcalá et al. (2019)
proPO-R	GGTGAGCATGAAGAAGAGCTGGA		
CTL-3	AAACCCTGGATTCGTCAA	171	Qin et al. (2018)
CTL-3	AAACCTTAGCTTAGAGTGGC		
PEN3	CACCCTTCGTGAGACCTTTG	141	Wang et al. (2010)
PEN3	AATATCCCTTTCCCACGTGAC		
PEN4	GCCCGTTACCCAAACCATC	106	Wang et al. (2010)
PEN4	CCGTATCTGAAGCAGCAAAGTC		
*β*-Actin	CCACGAGACCACCTACAAC	142	Wang, Chang & Chen (2007)
*β*-Actin	AGCGAGGGCAGTGATTTC		

A qPCR master mix was prepared (2X concentration = 7.5 ul/reaction) in one batch for all PCR reactions and store in aliquots at −20 °C. Each 2X reaction comprised 1.5 µL 10X Reaction Buffer, 0.75 µL 50 mM MgCl_2_, 0.3 µL 10 mM dNTPs, 0.75 µL EvaGreen® 20X (Biotium, Hayward, CA, USA), 0.1 µL Invitrogen® DNA Polymerase Recombinant (Life Technologies, Carlsbad, CA, USA) and 5.9 µL ultrapure water to 9.3 µL.

Before PCR, each aliquot of the 2X master mix was thawed, and 0.35 µl of each 10 µM primer (forward/reverse) was added to reach 10 µl. Then 10 µl of mix were placed in each plate well and 5 µl of the corresponding cDNA were added (15 µl per reaction).

### Histological analysis

After 28 days of treatment, three shrimp per treatment were collected and fixed in AFA Davidson’s solution for 48 h (111 ml glycerin, 222 ml formaldehyde (37–40%), 333 ml ethanol (96%), 233 ml filtered seawater, 100 glacial acetic acids) and treated according to Bell & Lighter (1988). Tissue sections were performed in 4 µm thick sections using a rotation microtome (Leica RM 2025) and stained with hematoxylin-eosin. Stained sections were examined with a compound microscope (Olympus BX-41, Nikon camera) and quantified based on B cell diameter using Image-Pro Plus 6.0 software. The degree of damage is defined according to Lightner (1996) (Table 2).

**Table 2:** Severity damage degrees in hepatopancreas cells.

**Severity degree**	**Description**
0	**No damage.** There was no obvious deformation of the hepatopancreatic tubules.
1	**Light damage (injuries less than 25% of the area).** Less tubular deformation and less cell detachment.
2	**Moderate damage (Injuries in 25 to 50% of the area).** A moderate number of deformed tubules (6 to 10 per organism) were observed. Hemocytes infiltration and nodules are seen.
3	**High damage (injuries in 50 to 75% of the area).** Many tubules are deformed (11 to 20 per organism). Moderate to severe melanization, cell detachment, tubular atrophy, and hemocytes nodule formation was observed.
4	**Severe damage (injuries over 75% of the area).** There are more deformed tubules (over 20 per organism). Severe melanization, necrosis, tubular atrophy, empty tubules, hemocyte nodules, and granulomas were observed.

### Statistical analysis

Statistical analysis was performed using the STATISTICA V6 program (StatSoft, Tulsa, OK, USA). Percentage values were normalized using the arcsine function before analysis. factorial-way ANOVA and *post hoc* Tukey’s comparison test were performed for expression, survival, WG, and FCR (*p* < 0.05). Histological damage data were analyzed using Kruskal-Wallis and *post hoc* Dunn’s test for multiple comparisons (*p* < 0.05).

## Results

### Toxicity and shrimp performance (bioassay 1)

There were no apparent signs of toxicity, and no mortality was recorded during treatment throughout the culture. Temperature, DO, and salinity values fluctuated between 25.3 and 27.5 °C, 5.5 and 6.7 mg/L, and 25 and 27 PSU, respectively. The shrimp performance values did not show significant differences between treatments (Table 3).

**Table 3:** Performance parameters of experimental shrimp fed with feed-AgNPs supplied at diferent frequencies.

Treatment	Initial mean weight (g)	Final mean weight (g)	WG^a^ (%)	SGR^b^	FCR^c^ (%)	Survival^d^ (%)
D1	4.8 ± 0.2	6.0 ± 0.3	24.0 ± 2.7	0.8 ± 0.1	2.9 ± 0.8	97 ± 2.9
D4	5.1 ± 0.0	6.1 ± 0.0	20.0 ± 0.4	0.7 ± 0.0	2.8 ± 0.1	100 ± 0.0
D7	4.8 ± 0.2	6.0 ± 0.3	24.2 ± 6.6	0.8 ± 0.2	2.4 ± 0.6	100 ± 0.0
Control	5.0 ± 0.1	6.1 ± 0.1	23.9 ± 2.8	0.8 ± 0.1	2.4 ± 0.3	100 ± 0.0


**Notes.**

a Weight gain (%) = [(final weight − initial weight)/initial weight] × 100.

b SGR = 100 (ln average final weight − ln average initial weight)/number of days.

c FCR = dry feed intake/wet weight gain.

d Survival (%) = [Final number of shrimp/Initial number of shrimp] × 100.

* There were no significant differences between the results of treatments.

### Antiviral activity assessment (bioassay 2)

After culture, the experimental shrimp were challenged against WSSV by oral route.

**Figure 2:**
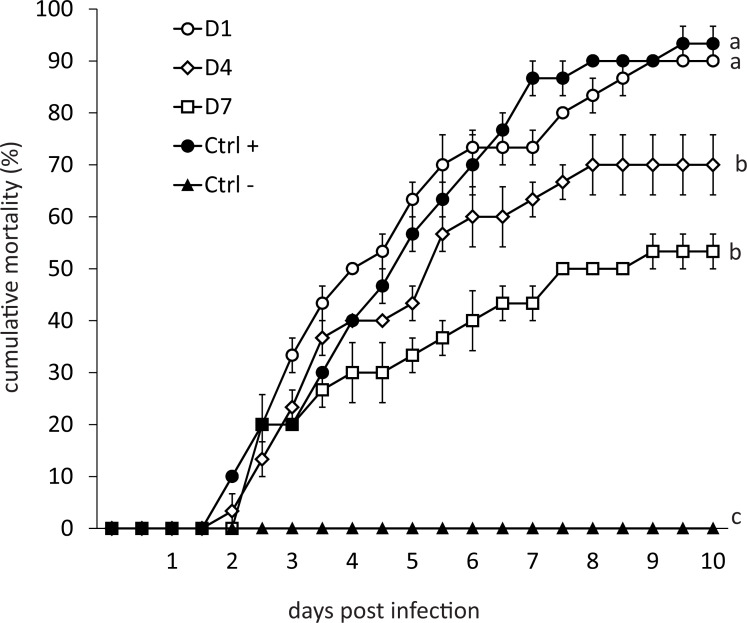
Cumulative mortality for ten days of shrimp challenged orally against WSSV. (D1) shrimp fed a ration of feed-AgNPs daily + WSSV. (D4) shrimp fed one ration of feed- AgNPs every 4th day + WSSV. (D7) shrimp fed with a ration of feed-AgNPs every 7th day + WSSV. (Control +) shrimp fed with AC + WSSV. (Ctrl -) shrimp fed with only AC. Different letters indicate signiffcant differences between treatments (*p* < 0.05).

Mortality of infected organisms was directly proportional to the frequency of feed-AgNPs supply (Fig. 2). Shrimp from the positive control (without AgNPs + WSSV) and treatment D1 achieved 93% and 90% mortality, respectively. Contrarily, treatments D4 and D7 achieved 70% and 53% mortality, respectively, and differed significantly from the positive control (*P* = 0.0098 and *P* = 0.0007, respectively). Negative control had no mortality.

### Transcriptional response of shrimp treated with feed-AgNPs in feed (bioassay 3)

#### Phagocytosis-activating protein gene expression

The expression of immune-related genes was assessed after 28 days of treatment.

**Figure 3:**
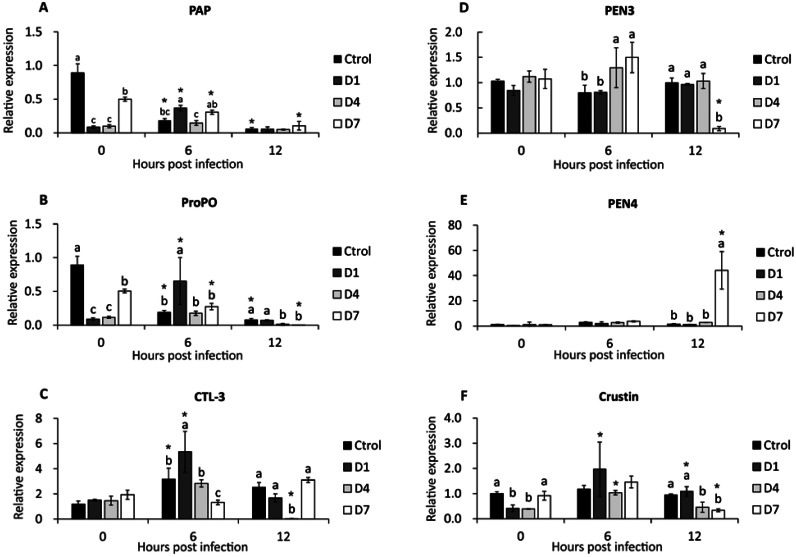
Transcriptional response of immune-related genes in shrimp, assessed before a WSSV infection (0 hpi) at 6 and 12 h post-challenge. (A) Phagocytosis activating protein (PAP). (B) Prophenoloxidase (ProPO). (C) C type lectin 3 (CTL-3). (D) Penaeidin 3 (PEN3). (E) Penaeidin 4 (PEN4). (F) Crustin. Shrimp were previously treated for 28 days with a dose of feed-AgNPs daily (D1) every 4 days (D4), every 7 days (D7), and with a control feed without AgNPs. Different letters indicate signiffcant differences between treatments at each sample time (*p* < 0.05). An asterisk (*) indicates signifficant differences with 0 hpi (*p* < 0.05).

PAP gene expression was down-regulated in control and D7 (6 and 12 hpi), and in D1, expression was up-regulated at 6 hpi (Fig. 3A). At 0 hpi, the control had the highest expression compared to the other treatments, and the expression of D7 was higher than D1 and D4. At 6 hpi, D1 reached the highest expression of PAP compared to control and D4. There was no difference between treatments at 12 hpi (Fig. 3A).

### Prophenoloxidase gene expression

After infection (6 and 12 hpi), the expression of the ProPO gene was down-regulated in control and D7. In contrast, D1 expression was up-regulated at 6 hpi (Fig. 3B).

Before infection, the control had the highest expression compared to the other treatments, and the expression of D7 was higher than D1 and D4. At 6 hpi, D1 reached higher expression than the other treatments. The control and D1 had higher expression than D4 and D7 at 12 hpi (Fig. 3B).

### C-type lectin 3 gene expression

At 6 hpi, CTL-3 expression was up-regulated in control, D1 and D4. Contrarily, in D4, expression was down-regulated at 12 hpi (Fig. 3C). Also, there was no significant difference between treatments before the challenge. At 6 hpi, treatment D1 had the highest expression and D7 had the lowest expression compared to the other treatments. At 12 hpi, the expression of treatment D4 was lower than the other treatments (Fig. 3C).

### Antimicrobial peptides gene expression (PEN3, PEN4, and Crustin)

There were no significant differences between pre-challenge treatments. At 6 hpi, D4 and D7 reached higher PEN3 expression than control and D1. At 12 hpi, PEN3 was down-regulated in D7 compared to other treatments (Fig. 3D).

PEN4 expression was up-regulated in D7 at 12 hpi (Fig. 3E). Also, there is no difference between the other sample time treatments.

Crustin expression was up-regulated at 6 and 12 hpi in D1 and 6 hpi in D4. In contrast, crustin expression in D7 at 12 hpi was down-regulated (Fig. 3F). However, there were no significant differences between treatments at 6 hpi. At 12 hpi, crustin expression was higher in control and D1 than in D4 and D7 (Fig. 3F).

### Histological analysis

**Figure 4:**
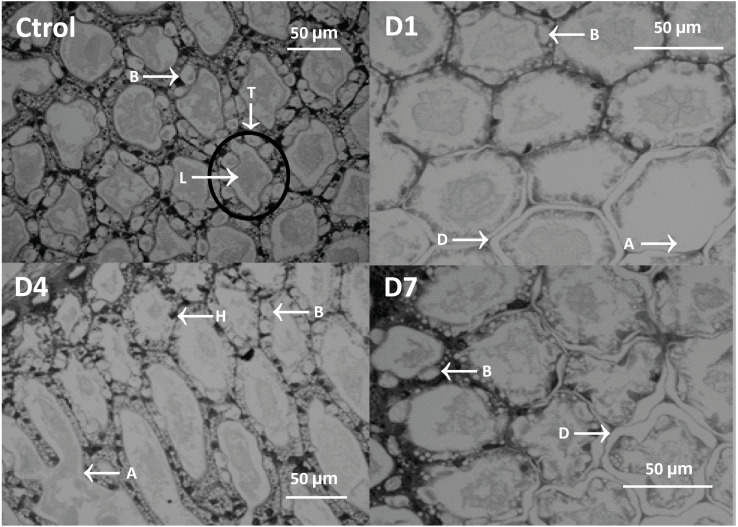
Micrograph of the hepatopancreas of shrimp fed with feed-AgNPs and control without AgNPs. B cells (B), tubules (T), lumen (L), hemocyte infiltration (H), detached cells (D), and atrophy (A).

Histological analysis showed atrophy, tubular delamination, and hemocyte infiltration in the hepatopancreas of shrimp from D1 and D4 treatments (Fig. 4).

**Figure 5:**
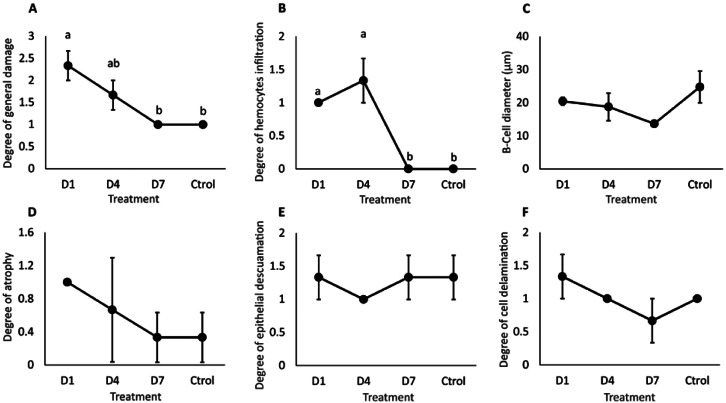
Semi-quantitative damage degree in the hepatopancreas of shrimp fed with feed- AgNPs. (A) General damage to the hepatopancreas. (B) Degree of hemocyte infiltration, (C) B-cell diameter, (D) cell atrophy, (E) epithelial desquamation, and (F) cell delamination. Results are presented as mean ± standard error. Different letters on the bars represent signfficant differences between treatments by Kruskal-Wallis analysis and Dunn’s test (*p* < 0.05).

General damage was directly proportional to the feed-AgNP doses supplied, reaching 2.3 and 1.6 damage degrees in D1 and D4, respectively. However, only D1 was significantly higher than D7 and control (1.0 damage degree for both) (Fig. 5A). Likewise, hemocyte infiltration was higher in treatments D1 and D4 (1.0 and 1.3 damage degrees, respectively). In contrast, no hemocyte infiltration was observed in control and D7 (Fig. 5B). No significant differences between treatments in B-cell diameter, cell atrophy, desquamation, and delamination (Figs. 5C–5F).

## Discussion

The incidence of WSSV in shrimp farms worldwide has encouraged research into new technologies to reduce its impact. Nanotechnology is a promising tool to help shrimp fight this pathogen. In this sense, silver nanoparticles can modulate the immune system of human cells, trigger an inflammatory response, and help suppress microorganisms (Tian et al., 2007; Dakal et al., 2016). Also, AgNPs have shown antiviral effects against pathogens such as HIV-1, hepatitis B virus, monkeypox virus, herpes simplex virus type I, tacaribe virus, H1N1 influenza A virus, canine distemper virus, and Rift Valley fever virus (Lu et al., 2008; Rogers et al., 2008; Baram-Pinto et al., 2009; Lara et al., 2010; Speshock et al., 2010; Mori et al., 2013; Bogdanchikova et al., 2016; Borrego et al., 2016). Promising results were also recorded in shrimp when AgNPs were injected (Juárez-Moreno et al., 2017) or supplied in feed (Romo-Quiñonez et al., 2020). Romo-Quiñonez et al. (2020) reported that AgNPs included in an 8-day daily ration significantly protected shrimp from WSSV infection by the oral route. However, no studies have been conducted on the regular administration of AgNPs for extended periods. Therefore, in this study, we provide the highest dose of AgNPs reported by Romo-Quiñonez et al. (2020), with three different cycles of 28 days.

After culture, the antiviral ability of AgNPs in the feed against WSSV was evaluated. The muscles from infected shrimp were liquefied and supplied to experimental shrimp, to simulate a natural infection. In this way, muscle tissue is divided into small fragments to reduce competition between organisms for larger particles. The results showed that protection against WSSV was provided by AgNPs and was directly proportional to the feed-AgNPs provided. This result suggests that the weekly application of feed-AgNPs suffices to protect shrimp from natural WSSV infection. However, an excess of silver can be counterproductive. This finding is similar to the other authors who reported that injection of 50, 200, and 2,000 ng AgNPs into 10 g shrimp prevented WSSV intramuscular infection. However, as shown in this study, mortality was higher with the highest dose of AgNPs, reaching 20%, 20% and 30% mortality, which differs from the 90% achieved in the control group without AgNPs (Juárez-Moreno et al., 2017).

When pathogens infect shrimp, reactive oxygen species (ROS) are produced as part of the phagocytic pathway (Song & Hsieh, 1994; Muñoz et al., 2000). Superoxide anion is a ROS that affects pathogens but can damage shrimp cell membranes. For these ROS not to harm shrimp, antioxidant-related enzymes such as SOD, GPx, and CAT reduces superoxide anion to H_2_O + O_2_ (Liu, Tseng & Cheng, 2007; Tian et al., 2011; Trasviña Arenas et al., 2013). A retrospective study concluded that metals can induce or inhibit antioxidant enzymes, depending on species, kind of metal, amount, and exposure time (Frías-Espericueta et al., 2022). More specifically, AgNPs interact with copper-zinc-superoxide dismutase to induce structural changes affecting SOD and CAT functions (Zhang et al., 2015; Liu, Worms & Slaveykova, 2020). This phenomenon includes crustaceans (Walters et al., 2016). AgNPs interact with antioxidant systems that promote lipid peroxidation (Carlson et al., 2008). Physiological changes induced by exposure to aqueous silver have been reported in the crustacean *Cambarus diogenes* (Grosell et al., 2002). Therefore, we can hypothesize different physiological and metabolic responses in shrimp organs by exposure to nanometals. This phenomenon could explain the high mortality of shrimp treated with the once-daily feed-AgNPs, suggesting that excessive silver intake can lead to adverse effects.

Assessing immune system gene expression by quantitative PCR is a tool for assessing the immunity of shrimp under specific conditions (Pourmozaffar, Hajimoradloo & Miandare, 2017; Soto-Alcalá et al., 2019; Han et al., 2021). The shrimp immune system is primarily mediated by hemocytes, which function similarly to vertebrate white blood cells and are involved in defense mechanisms against pathogens. (Söderhäll & Smith, 1983; Sritunyalucksana & Söderhäll, 2000). Several mechanisms stimulate oxidative metabolites, melanin production, and activate the ProPO system (Sritunyalucksana & Söderhäll, 2000), which stimulates phagocytosis (Söderhäll, Aspán & Duvic, 1990). The ProPO system and phagocytosis are two fundamental defense mechanisms in crustaceans against pathogens (Sung, Yang & Song, 1996; Vazquez et al., 2009). Phagocytosis-activating proteins are associated with the phagocytosis pathway and their gene expression is increased when shrimp are exposed to WSSV (Deachamag et al., 2006).

In this study, the expression of PAP and ProPO was down-regulated in the treatment with AgNPs compared to the control group after 28 days of treatment. Contrarily, PAP and ProPO transcriptional responses to WSSV were higher in shrimp with greater silver supply (D1) after 6 hpi. Romo-Quiñonez et al. (2020) showed that PAP and ProPO gene expression was unaffected in shrimp fed AgNPs at 6, 12, 24, and 48 h after feeding with AgNPs. Short-term exposure (30 min) of mussel (*Mytilus galloprovincialis*) hemocytes to AgNPs did not affect their phagocytic capacity (Auguste et al., 2018), and AgNPs injected into shrimp did not alter hemocyte numbers in white shrimp (Juárez-Moreno et al., 2017). These findings suggest that short-term exposure to AgNPs does not interfere with immune system gene expression.

Silver also appears to inhibit basic defense mechanisms in shrimp in a long term. Besides, PAP and ProPO gene expression after culture were consistent with the mortality observed in the challenge bioassay (Fig. 2).

Lectins play essential roles in many biological processes, such as molecular effectors, cell signaling, and pathogen recognition (Wang & Wang, 2013). In this study, feed-AgNPs did not affect the expression of CTL-3 before WSSV infection; however, the CTL-3 gene was up-regulated after infection in shrimp fed with daily AgNPs. Lectins enhance phagocytosis and sometimes prevent WSSV (Zhao et al., 2009; Chen et al., 2013); however, in other cases, lectins promote infection (Dai et al., 2016; Huang et al., 2022). These results of this study suggest that CTL-3 is not involved in the defense mechanism against WSSV.

Antimicrobial peptides (AMPs) are components in many organisms’ pathogen defense mechanisms (Brown & Hancock, 2006). The penaeidins are penaeid shrimp-specific AMPs (Destoumieux et al., 1997), and several penaeidins species and crustin have shown activity against WSSV (García et al., 2009). However, paradoxically, some expressions of penaeidins are down-regulated after WSSV infection (Jeswin et al., 2013; Xue et al., 2013; Zhang et al., 2018). This study showed that PEN3 and PEN4 expressions were not affected by feed-AgNPs before WSSV infection; howevee, 12 h after infection, PEN3 expression was down-regulated and PEN4 expression was up-regulated. Transcriptional response patterns of PEN3 and PEN4 genes suggest that exposure to AgNPs could regulate expression. The cellular damage recorded by histology in this study showed a direct relationship between silver supply and the level of hepatopancreatic cell damage. Multiple lesions in the hepatopancreas were recorded in shrimp fed daily AgNPs. Contrarily, a weekly supply of feed-AgNPs appears to be a better strategy to protect shrimp from WSSV in culture. Ribeiro et al. (2014) found acute toxic effects on *Daphnia magna* following 21-day exposure to AgNPs at concentrations above 1 µg/L, affecting feeding rates and reproduction. Chávez-Sánchez et al. (2020) observed significant changes in shrimp muscle when AgNPs were directly released into the hemocoel. However, this study recorded milder cellular damage after 28 days of exposure, possibly due to the internalization of AgNPs into shrimp cells from the outside through the digestive tract. Also, no mortality was recorded during the 28-day culture period. Therefore, the cellular damage recorded in some treatments is not fatal; excess AgNPs made shrimp vulnerable to WSSV.

In WSSV-infected shrimp, mortality may reach 100% three to 10 days after infection (Liu, Söderhäll & Jiravanichpaisal, 2009). Recently published results on using plant-derived compounds against WSSV have been published, but shrimp mortality was only delayed in time compared to the positive control group (Muliani et al., 2021; Hu et al., 2022; Shan et al., 2022). Medina-Félix et al. (2014) challenged shrimp *L. vannamei* treated with feed supplemented with unicellular microalgae *Dunaliella* sp. against WSSV, reaching mortality significantly lower in treated shrimp (20%) compared with the control group (44%). However, measurements were performed only once (on the sixth day after infection). Therefore, it is not clear what will happen to shrimp during the critical days 9–10. In this research, the application of AgNPs resulted in 53% mortality at day 10 post-infection (which is a critical time limit for shrimp mortality in the WSSV case), compared with 93% in the positive control group. The most important revelation, however, was that on day 10, the mortality curve plateaued, indicating the end of shrimp mortality (Fig. 2). This result indicates that AgNPs (Argovit-4®) are promising in reducing shrimp mortality caused by WSSV. Therefore, the dose of AgNPs should be further optimized to determine the best AgNPs’ efficiency.

Finally, the results showed that the antiviral activity of AgNPs (Argovit-4®) was more effective when supplied for a long time, possibly because, in this case, the silver exerted its antimicrobial effect while the shrimp also eliminated the excess metallic silver on their own.

In conclusion, this study showed that supplementation of 1,000 µg AgNPs (Argovit-4®) per gram of feed did not interfere with shrimp growth and prevented WSSV infection when AgNPs were supplied once a week. Frequent feedings (every day or every four days) can lead to poor results. However, transcriptional responses of immune-related genes were affected. Whether this affectation makes the shrimp vulnerable to other pathogens is unknown and remains to be investigated. Also, further optimization of the AgNPs concentration in the feed and the feeding cycle could be more effective in preventing WSSV infection.

##  Supplemental Information

10.7717/peerj.14231/supp-1 File S1 ANOVA of final mortality after 10 days of the WSSV infection Mortality was recorded in each replicate from treatments every 12 hours for ten days. The cumulative mortality at the end of the experiment was normalized by arcsine function and analyzed by one-way ANOVA and a Tukey test using the Statistic program (STATISTICA 7.0).

10.7717/peerj.14231/supp-2 File S2 ANOVA of the transcriptional response to WSSV Transcriptional response of experimental shrimp after experimental infection with WSSV and at 9 and 12 hours post-infection. Data are represented as the relative expression of each gene in all treatments. The data were analyzed by two-way ANOVA using the time and treatment factors. Tukey test was interpreted as differences between treatment each time and the difference between before and after infection.

## Additional Information and Declarations

### Data Availability

The following information was supplied regarding data availability: The mortality and gene expression raw data are available in the Supplementary Files.
